# Characterizing primary care in breast cancer survivors after completion of cancer therapy

**DOI:** 10.1007/s10552-026-02230-4

**Published:** 2026-08-19

**Authors:** Kendall Kay, Yancen Pan, Jessica Li, Lan Zhang, Vanessa N Torres, Yelba Castellon-Lopez, Meng-Han Tsai, Geetanjali D Datta, Jennifer Henry, Cody Ramin

**Affiliations:** 1 Cancer Research Center for Health Equity, Cedars-Sinai Medical Center, 6500 Wilshire Blvd, Los Angeles, CA 90048 USA; 2 Cedars-Sinai Cancer, Cedars-Sinai Medical Center, Los Angeles, CA USA; 3 Cancer Prevention, Control, & Population Health Program, Georgia Cancer Center, Augusta University, Augusta, GA USA; 4 Georgia Prevention Institute, Augusta University, Augusta, GA USA; 5 Links for Life, Bakersfield, CA USA

**Keywords:** Breast cancer, Cancer survivorship, Primary care, Survivorship care, Healthcare access

## Abstract

**Purpose:**

Despite the potential for primary care to optimize comorbidity management among breast cancer survivors, engagement with primary care providers (PCPs) remains low. This study comprehensively examined PCP-led care after breast cancer treatment to identify survivors who may benefit from targeted interventions to improve primary care engagement.

**Methods:**

We identified 1,582 posttreatment breast cancer survivors from the U.S. Behavioral Risk Factor Surveillance System survey. Weighted multivariable-adjusted logistic regression models were used to examine associations of sociodemographic, lifestyle, clinical, and survivorship-related factors with PCP-led care after cancer treatment.

**Results:**

Overall, 72% of survivors reported receiving PCP-led follow-up (64% among those aged < 65 years; 75% among those aged ≥ 65 years), with survivors aged < 65 years having significantly lower odds of PCP-led care than those aged ≥65  years (OR: 0.63, 95% CI: 0.41–0.98). Racial and ethnic differences were also evident with lower odds of receiving primary care among Non-Hispanic (NH)-Black and NH-Asian survivors compared with NH-White survivors (OR: 0.49, 95% CI: 0.24–0.97; OR: 0.33, 95% CI: 0.11–0.97, respectively), with particularly lower odds among younger Hispanic survivors (OR: 0.13, 95% CI: 0.03–0.49). Geographic variation across regions was also observed (South OR: 0.42, 95% CI: 0.25–0.72; West OR: 0.60, 95% CI: 0.37–0.96, compared with Midwest). Survivors with a history of myocardial infarction further had lower odds of PCP-led care (OR: 0.40, 95% CI: 0.20–0.80).

**Conclusion:**

Gaps in PCP-led care after breast cancer treatment underscore the importance of improving access and support for PCP-led engagement throughout survivorship. Future studies are needed to examine barriers to PCP-led care among younger survivors, NH-Black and NH-Asian survivors, and those with heart disease, and to characterize region-specific differences in PCP-led care across the U.S.

**Supplementary Information:**

The online version contains supplementary material available at https://doi.org/10.1007/s10552-026-02230-4.

## Introduction

In 2025, there were an estimated 4.3 million breast cancer survivors in the United States (U.S.), and this number is projected to increase to 5.3 million by 2035 [1]. Despite increasing survival rates, many breast cancer survivors continue to experience a broad range of challenges after treatment, including comorbidities, as well as psychological, social, and quality of life concerns [2, 3]. As a result, most breast cancer survivors require long-term follow-up care for prevention and management of these challenges, including an increased burden of comorbidities, such as weight gain, diabetes, hypertension, dyslipidemia, and cardiovascular disease [3–6]. Breast cancer survivors often become “lost in transition” from oncology to primary care at the end of active treatment, resulting in a lack of high-quality comprehensive follow-up care, even 20 years after the National Academies of Science, Engineering, and Medicine (NASEM; formerly the Institute of Medicine) recommended survivorship care plans to improve these care transitions [7]. Although survivorship care plans have been endorsed by the American Society of Clinical Oncology (ASCO), the American Cancer Society (ACS), and the National Comprehensive Cancer Network (NCCN), these plans have remained widely underutilized, which may contribute to gaps in survivorship care coordination [8]. Notably, the Commission on Cancer no longer requires the delivery of survivorship care plans as a component of its accreditation standards due to underutilization and inconsistent evidence that these plans improve health outcomes [9–11].

Consistent with these gaps in survivorship care coordination, prior studies suggest that primary care engagement after cancer treatment remains limited. A recent study from 2024 found that primary care providers (PCPs) were engaged in survivorship care for less than 50% of cancer survivors beyond 5 years postdiagnosis, and even among those who reported high initial primary care engagement, many experienced declining engagement over time [12]. Additional recent research from the iCanCare study has further demonstrated that PCP-led survivorship care is low among long-term breast cancer survivors [13] and that PCP engagement declines over time [14]. In addition, recent research also reported that only 24% of cancer survivors were comfortable receiving survivorship care from a PCP instead of an oncology provider [15] and that preference for PCP vs. oncology-directed survivorship care differs by race, ethnicity, and educational attainment with Black and Asian survivors and those with a high school education or less more likely to prefer receiving preventive and comorbidity care from their oncologists rather than PCPs [16]. However, cancer survivors engaging with PCP-led care are more likely to receive recommended preventive care than those with oncology-based follow-up [17]. In addition, prior studies on PCP-led models of cancer survivorship care have demonstrated that they may lower costs for survivors and healthcare systems [18, 19]. As such, the National Cancer Institute recently identified supporting care transition from oncology to primary care as a key research priority in the 2024 National Standards for Cancer Survivorship [20, 21].

Few studies have evaluated survivorship care patterns (e.g., type of providers) among breast cancer survivors and most were conducted several decades ago [14, 17, 22–26]. Existing studies have been largely descriptive, focused on oncology-led care, and have examined only a limited number of sociodemographic (e.g., insurance status, income, and race/ethnicity) and clinical (e.g., time since cancer diagnosis) factors that may be associated with PCP-led care [17, 22, 23]. Other previous studies descriptively examined patterns of survivorship care and factors associated with patient-reported PCP engagement and communication, but did not examine factors associated with PCP-led care after cancer treatment [14, 24–26]. Therefore, a comprehensive assessment of factors associated with PCP-led care in breast cancer survivors is needed.

For this study, we aimed to identify sociodemographic, lifestyle, clinical, and survivorship-related factors associated with PCP-led care in U.S. breast cancer survivors after the completion of cancer treatment. Understanding factors associated with PCP-led care may help to identify survivors who are less likely to receive this care and guide targeted interventions to increase the number of breast cancer survivors successfully engaging with PCPs.

## Methods

### Data source

Our study utilized data from the Behavioral Risk Factor Surveillance System (BRFSS) survey, a cross-sectional survey administered by the U.S. Centers for Disease Control and Prevention (CDC). The survey contains the annual standard core and biannual rotating core questions utilized by every state, which cover a broad range of sociodemographic, chronic health, and lifestyle topics. The survey also includes optional modules, such as the cancer survivorship module, that states may opt in to administer. The BRFSS questionnaire was developed in collaboration between the CDC and public health departments of the U.S. states, the District of Columbia, Puerto Rico, and the U.S. Virgin Islands. All participant responses were self-reported [27].

### Study population

Our analysis included data from the 2012, 2017, 2019, and 2021 BRFSS surveys and was restricted to participants from 13 U.S. states and territories that administered the cancer survivorship module (n = 108,639). These four recent survey years were selected for their inclusion of questions on cancer survivorship and lifestyle factors. We further restricted our analytic population to women who reported a breast cancer diagnosis (n = 2,285; 22 males were excluded due to small numbers) and who were not currently receiving treatment for cancer, including surgery, radiation therapy, or chemotherapy (n = 1,613). We excluded participants with missing information on the type of clinician providing the majority of their survivorship care (n = 31; < 2%). This resulted in a final analytic cohort of 1,582 breast cancer survivors for analysis (Supplementary Fig. 1).

### Ascertainment of PCP-led care

Our outcome of interest was PCP-led care after cancer treatment completion. PCP-led care was defined using the following question from the cancer survivorship module, which ascertains information on the type of doctor participants see most often for regular health care (e.g., annual exams, treatment for general illness) by asking, “What type of doctor provides the majority of your health care?” [27] Responses were collapsed into two groups: (1) PCP (family practitioner or general practitioner/internist) and (2) non-PCP (cancer surgeon, general surgeon, gynecologic oncologist, plastic/reconstructive surgeon, medical oncologist, radiation oncologist, urologist, other). The full distribution for type of doctor is provided in Supplementary Table 1.

### Ascertainment of sociodemographic factors

Sociodemographic factors included current age, race and ethnicity, education level, annual household income, current health insurance status, and U.S. region. Current age was categorized into < 50 years, 50- < 60 years, 60- < 70 years, 70- < 80 years, and ≥ 80 years. We also examined age as a binary variable categorized into < 65 years and ≥ 65 years as a proxy for Medicare eligibility, which may represent differences in healthcare access [28]. Race and ethnicity were defined as non-Hispanic (NH)-White, NH-Black, NH-Asian, Hispanic, and NH-other race. We combined American Indian/Alaska Native women with those who reported NH-other race due to small numbers. Education level was defined as high school or less, some college or technical school, and completed college or technical school. Annual household income was categorized into < $25,000, $25,000- < $50,000, and ≥ $50,000. Current health insurance was classified as having any form of health insurance at the time of survey completion (yes, no). Region was categorized into Midwest (Indiana, Michigan, Nebraska, South Dakota, and Wisconsin), South (Delaware and Georgia), and West (Hawaii, Montana, New Mexico, Utah, and Wyoming) using classification guidelines from the U.S. Census Bureau [29]. Respondents from Puerto Rico were combined with the Southern region due to small numbers. Our analytic population did not include any participants from the Northeast due to small sample size.

### Ascertainment of lifestyle and clinical factors

Lifestyle factors included fruit and vegetable consumption, smoking status, alcohol consumption, body mass index (BMI), and physical activity. These were examined due to their contribution to the burden of cancer and cancer survivorship [30, 31]. Participants reported the frequency per day that they consumed fruits, including 100% fruit juice, and vegetables, including dark green vegetables, potatoes (excluding fried potatoes), and other vegetables. We categorized fruit and vegetable consumption into consuming fruits or vegetables < 5 or ≥ 5 times per day, which was decided based on the ACS cancer prevention and survivorship recommendations [31, 32]. Smoking status was defined as ever (current, former) and never smokers. Alcohol consumption was categorized as excessive or non-excessive. Excessive alcohol consumption was defined per ACS recommended guidelines as having an average of more than one alcoholic drink per day across the last 30 days or having an episode of drinking four or more alcoholic beverages on one occasion during the past 30 days (binge drinking) [31]. BMI was categorized into underweight or normal weight (< 25 kg/m^2^), overweight (25- < 30 kg/m^2^), and obese (≥ 30 kg/m^2^). Physical activity was assessed by asking if respondents participated in any form of physical activity or exercise during the past month (yes, no). Comorbidities were based on those available in the BRFSS survey and included history of myocardial infarction (yes, no), angina or coronary heart disease (CHD; yes, no), stroke (yes, no), asthma (yes, no), chronic obstructive pulmonary disease (COPD), emphysema, or chronic bronchitis (yes, no), depressive disorder (yes, no), kidney disease (yes, no), diabetes (excluding gestational diabetes; yes, no), and arthritis, rheumatoid arthritis, gout, lupus, or fibromyalgia (yes, no). We also created a composite variable to represent comorbidity burden defined as a history of one or more comorbid conditions, which included any of the comorbidities listed above. These comorbidities were included due to differences in follow-up care needs for those with cardiometabolic disease and comorbidity burden [4].

### Ascertainment of survivorship care factors

The cancer survivorship module contained several questions on survivorship care, including whether respondents had ever received (1) a written summary of their cancer treatments (yes, no) and (2) instructions regarding who to return to for medical checkups after their treatment completion (yes, no). Health insurance during cancer treatment was defined as having health insurance that covered all or part of the participants’ cancer treatment (yes, no). Age at cancer diagnosis was reported by having participants answer the question “At what age were you told that you had cancer?” Responses were collapsed into < 65 years and ≥ 65 years to examine differences by age and as a proxy for Medicare eligibility to represent potential differences in healthcare access. We also assessed years since diagnosis, categorized as 0–1 year, 2–3 years, 4–5 years, and ≥ 6 years. Years since diagnosis was calculated by subtracting age at the time of the first cancer diagnosis from current age at survey completion.

### Statistical analysis

Characteristics of breast cancer survivors were summarized overall and by PCP-led care using weighted and unweighted frequencies and percentages. We used logistic regression to estimate odds ratios (ORs) and 95% confidence intervals (CIs) for the associations between sociodemographic, lifestyle, clinical, and survivorship-related factors with PCP-led care. Complex sampling procedures were accounted for in all analyses through the inclusion of sample weights, strata, and clusters. Weights were scaled proportionally for each year based on their contribution to the final analytic sample size [27]. Due to small numbers, we combined singleton strata with others from the same year and state to reduce potential underestimation in variance calculations [33, 34]. We conducted a series of multivariable-adjusted models: Model 1 adjusted for age (< 50, 50- < 60, 60- < 70, 70- < 80, ≥ 80 years), Model 2 adjusted for covariates in Model 1 plus race and ethnicity (non-Hispanic White, non-Hispanic Black, non-Hispanic Asian, Hispanic, and non-Hispanic other race), educational attainment (high school or less, some college or technical school, college or technical school graduate), and annual household income (< $25,000, $25,000–< $50,000, ≥ $50,000), and Model 3 adjusted for covariates in Model 2 plus health insurance during cancer treatment (yes, no). ORs were similar across Models 2 and 3; therefore Model 2 is presented as our main model. Tests for trend were estimated with ordinal variables for age, annual household income, BMI, and years since diagnosis. Multivariable-adjusted models were also stratified by (1) age (< 65, ≥ 65 years) to examine whether results differed by age group and to assess potential differences in healthcare access using age 65 years as a proxy for Medicare eligibility [28], and (2) by years since diagnosis (< 5, ≥ 5 years). We further conducted a sensitivity analysis by (1) restricting our models to those with current health insurance (n = 1,545; > 97%) and (2) adjusting multivariable models for survey year (2012, 2017, 2019, 2021), however, results were materially unchanged and are therefore not presented. SAS version 9.4 (Cary, North Carolina) was used for all analyses. Two-sided p-values < 0.05 were considered statistically significant.

## Results

### Population characteristics

Among 1,582 breast cancer survivors, most (71.68%) were ≥ 65 years old, NH-White (82.49%, 4.93% Hispanic, 4.87% Black, and 3.54% Asian), and had health insurance that covered some or all of their cancer treatment costs (95.01%), history of one or more comorbidities (71.68%), and current health insurance (97.66%) (Table 1). Over 70% were long-term breast cancer survivors (≥ 6 years since diagnosis), half (50.51%) reported receiving a summary of their cancer treatments, and 77.56% received instructions regarding who to return to for check-ups after completion of cancer therapy. Approximately 72% of breast cancer survivors reported receiving PCP-led care (64.22% among those aged < 65 years, 74.78% among those aged ≥ 65 years). Survivors with PCP-led care were more likely to be older, NH-White, living in the Midwest, have one or more comorbidities, and to be long-term survivors compared to those without PCP-led care (Supplementary Table 1).

**Table 1:** Overall characteristics of breast cancer survivors in the Behavioral Risk Factor Surveillance System (n = 1,582)

Respondent characteristics	Unweighted			Weighted	
	n	%		n	%
Age, years					
< 50	64	4.05		9311	5.85
50– < 60	189	11.95		21,503	13.51
60– < 70	475	30.03		50,800	31.91
70– < 80	520	32.87		49,774	31.26
≥ 80	322	20.35		25,860	16.24
No response	12	0.76		1957	1.23
Age (binary), years					
< 65	436	27.56		53,815	33.80
≥ 65	1134	71.68		103,433	64.97
No response	12	0.76		1957	1.23
Race & ethnicity					
Non-Hispanic White	1305	82.49		122,479	76.93
Non-Hispanic Black	77	4.87		14,633	9.19
Non-Hispanic Asian	56	3.54		4763	2.99
Hispanic	78	4.93		14,295	8.98
Non-Hispanic other race	66	4.17		3035	1.91
Education level					
High school or less	505	31.92		59,580	37.42
Some college/technical school	515	32.55		59,344	37.28
Graduated college/technical school	562	35.52		40,280	25.30
Annual income					
< $25,000	362	22.88		37,373	23.47
$25,000–< $50,000	394	24.91		34,504	21.67
≥ $50,000	545	34.45		58,051	36.46
No response	281	17.76		29,278	18.39
Region					
Midwest	870	54.99		92,790	58.28
South & territories	256	16.18		46,235	29.04
West	456	28.82		20,180	12.68
Health insurance (current)^a^					
No	29	1.83		5702	3.58
Yes	1545	97.66		152,643	95.88
No response	8	0.51		859.84	0.54
BMI, kg/m^2^					
< 25	513	32.43		49,512	31.10
25–< 30	525	33.19		60,167	37.79
≥ 30	456	28.82		40,700	25.56
No response	88	5.56		8826	5.54
Smoking status					
Never smoked	958	60.56		97,773	61.41
Current/former smoker	615	38.87		60,679	38.11
No response	9	0.57		753.09	0.47
Alcohol consumption^b^					
Non-excessive	1468	92.79		144,573	90.81
Excessive	105	6.64		12,826	8.06
No response	9	0.57		1806	1.13
Fruit & vegetable consumption					
< 5 times/day	1206	76.23		120,805	75.88
≥ 5 times/day	302	19.09		32,121	20.18
No response	74	4.68		6279	3.94
Exercised in the past month^c^					
No	484	30.59		50,729	31.86
Yes	1096	69.28		108,380	68.08
No response	2	0.13		96.09	0.06
History of one or more comorbid conditions^d^					
No	437	27.62		40,150	25.22
Yes	1134	71.68		118,581	74.48
No response	11	0.70		474.24	0.30
History of myocardial infarction					
No	1486	93.93		150,018	94.23
Yes	93	5.88		8697	5.46
No response	3	0.19		490.82	0.31
History of angina or CHD					
No	1461	92.35		146,292	91.89
Yes	107	6.76		11,167	7.01
No response	14	0.88		1747	1.10
History of stroke					
No	1481	93.62		149,279	93.77
Yes	99	6.26		9548	6.00
No response	2	0.13		377.55	0.24
History of diabetes					
No	1292	81.67		132,324	83.12
Yes	289	18.27		26,697	16.77
No response	1	0.06		184.18	0.12
Age at cancer diagnosis, years					
< 65	1151	72.76		117,162	73.59
≥ 65	385	24.34		35,830	22.51
No response	46	2.91		6212	3.90
Years since cancer diagnosis					
0–1	99	6.26		8523	5.35
2–3	138	8.72		12,549	7.88
4–5	136	8.60		12,972	8.15
≥ 6	1145	72.38		116,116	72.93
No response	64	4.05		9045	5.68
Health insurance during cancer treatment^e^					
No	65	4.11		5367	3.37
Yes	1503	95.01		150,915	94.79
No response	14	0.88		2923	1.84
Receipt of treatment summary					
No	694	43.87		62,865	39.49
Yes	799	50.51		89,801	56.41
No response	89	5.63		6539	4.11
Receipt of return instructions					
No	318	20.10		33,348	20.95
Yes	1227	77.56		121,529	76.34
No response	37	2.34		4328	2.72

*BMI* body mass index, *CHD* coronary heart disease

^a^Health insurance (current) was evaluated by asking participants if they had any form of healthcare coverage, prepaid plans such as HMOs, or government plans such as Medicare

^b^Excessive alcohol consumption was defined as a daily average of more than one alcoholic drink or an episode of drinking four or more alcoholic drinks in the last month

^c^Exercising in the past month includes 1 or more instance of doing any type of exercise in the past 30 days

^d^History of one or more comorbid conditions is defined as having myocardial infarction, angina or coronary heart disease, stroke, asthma, chronic obstructive pulmonary disease, emphysema, chronic bronchitis, depressive disorder, kidney disease, diabetes, arthritis, rheumatoid arthritis, gout, lupus, or fibromyalgia

^**e**^Health insurance during cancer treatment was evaluated by asking participants whether they had health insurance that paid for all or part of their cancer treatment

### Associations between sociodemographic factors and PCP-led care

**Fig. 1:**
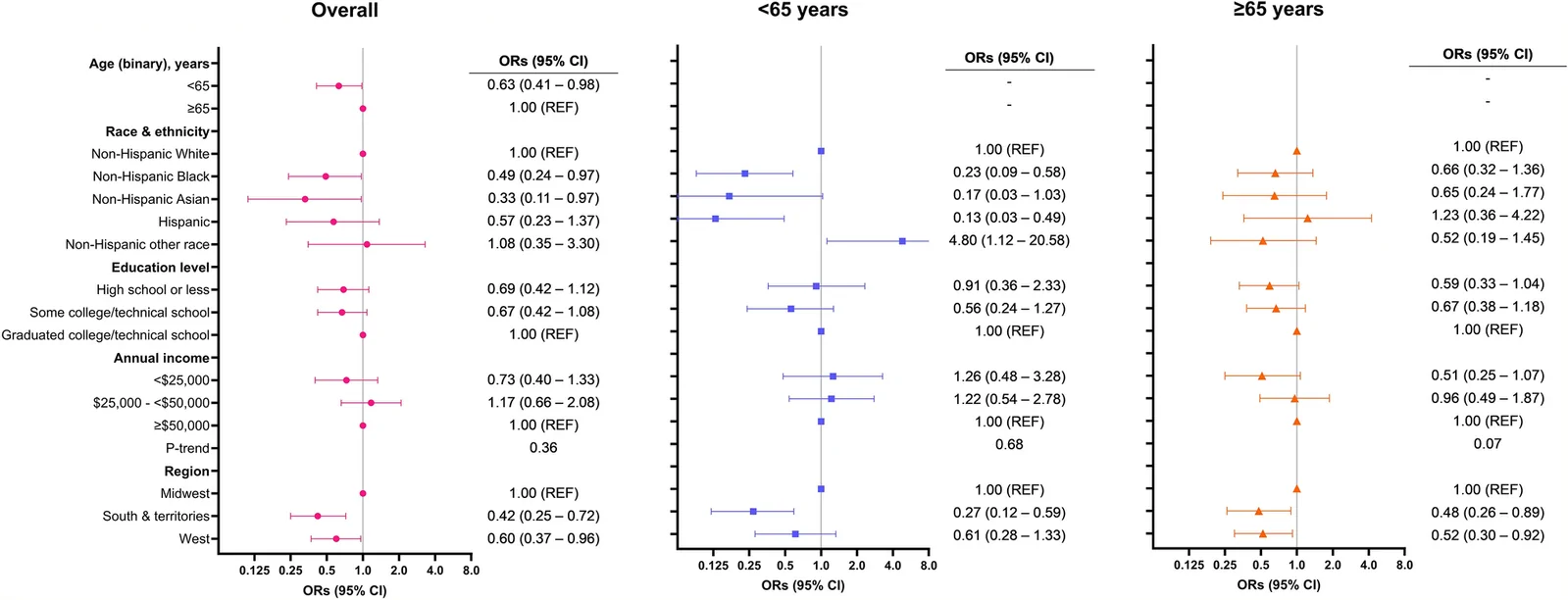
Associations between sociodemographic factors and PCP-led survivorship care among breast cancer survivors, overall and stratified by age (n = 1,582)^a^. ^a^Models adjust for age (< 50, 50– < 60, 60– < 70, 70– < 80, ≥ 80 years), race and ethnicity (non-Hispanic White, non-Hispanic Black, non-Hispanic Asian, Hispanic, and non-Hispanic other race), educational attainment (high school or less, some college or technical school, college or technical school graduate), and annual income (< $25,000, $25,000– < $50,000, ≥ $50,000)

Overall, we found that age, race and ethnicity, and U.S. region were significantly associated with PCP-led care (Fig. 1, Supplementary Table 2). Survivors aged < 65 years had significantly lower odds of receiving PCP-led survivorship care compared to those aged ≥ 65 years (OR: 0.63, 95% CI 0.41–0.98) (Fig. 1). Compared to NH-White survivors, NH-Black survivors (OR: 0.49, 95% CI 0.24–0.97) and NH-Asian survivors (OR: 0.33, 95% CI 0.11–0.97) were significantly less likely to receive PCP-led care, particularly among those aged < 65 years (NH-Black OR: 0.23, 95% CI 0.09–0.58; NH-Asian OR: 0.17, 95% CI 0.03–1.03). Among this younger age group (< 65 years), Hispanic survivors were also less likely to receive PCP-led care when compared with NH-White survivors (OR: 0.13, 95% CI 0.03–0.49). Compared to survivors from the Midwest, survivors from the South (OR: 0.42, 95% CI 0.25–0.72) and West (OR: 0.60, 95% CI 0.37–0.96) also had lower odds of PCP-led care. There were no significant associations between education level, annual income, and PCP-led care. Results were largely unchanged overall when restricted to survivors with current health insurance (Supplementary Table 3). For models stratified by time since diagnosis, results for race and ethnicity were similar but stronger in magnitude among recent survivors (< 5 years postdiagnosis) and attenuated among long-term survivors (≥ 5 years postdiagnosis) (Supplementary Table 3).

### Associations between lifestyle and clinical factors and PCP-led care

**Fig. 2:**
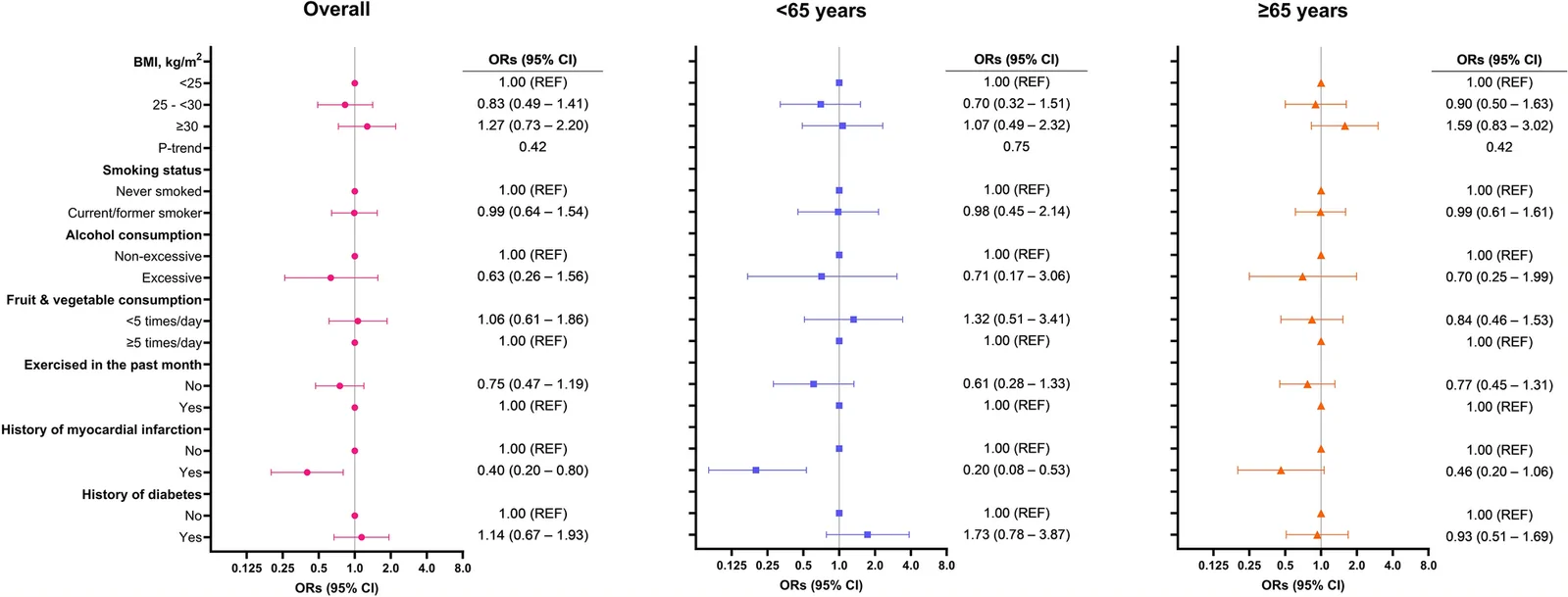
Associations between lifestyle and clinical factors and PCP-led survivorship care among breast cancer survivors, overall and stratified by age (n = 1,582)^a,b,c^. Abbreviations: BMI, body mass index. ^a^Models adjust for age (< 50, 50– < 60, 60– < 70, 70– < 80, ≥ 80 years), race and ethnicity (non-Hispanic White, non-Hispanic Black, non-Hispanic Asian, Hispanic, and non-Hispanic other race), educational attainment (high school or less, some college or technical school, college or technical school graduate), and annual income (< $25,000, $25,000– < $50,000, ≥ $50,000). ^b^Excessive alcohol consumption was defined as a daily average of more than one alcoholic drink or an episode of drinking four or more alcoholic drinks in the last month. ^**c**^Exercising in the past month includes 1 or more instance of doing any type of exercise in the past 30 days

Lifestyle factors, including BMI, smoking status, alcohol consumption, fruit and vegetable consumption and exercise, and comorbidities, were overall not significantly associated with PCP-led care (Fig. 2, Supplementary Tables 4 and 5). However, survivors with a history of myocardial infarction had significantly lower odds of PCP-led care (OR: 0.40, 95% CI 0.20–0.80), particularly among those aged < 65 years (OR: 0.20, 95% CI 0.08–0.53) (Fig. 2, Supplementary Table 4). Overall, results were similar when restricting to survivors with current health insurance (Supplementary Table 6). For models stratified by time since diagnosis, patterns of association mostly remained for lifestyle and clinical factors. However, we observed that recent survivors (< 5 years since diagnosis) with lower fruit and vegetable consumption and without exercise in the past month were less likely to have engaged with PCP-led care (OR: 0.22, 95% CI 0.10–0.50; OR: 0.39, 95% CI 0.17–0.89, respectively) (Supplementary Table 6). In addition, long-term survivors (≥ 5 years since diagnosis) with myocardial infarction were significantly less likely to have PCP-led survivorship care (OR: 0.27, 95% CI 0.13–0.59) (Supplementary Table 6).

### Associations between survivorship care factors and PCP-led care

**Fig. 3:**
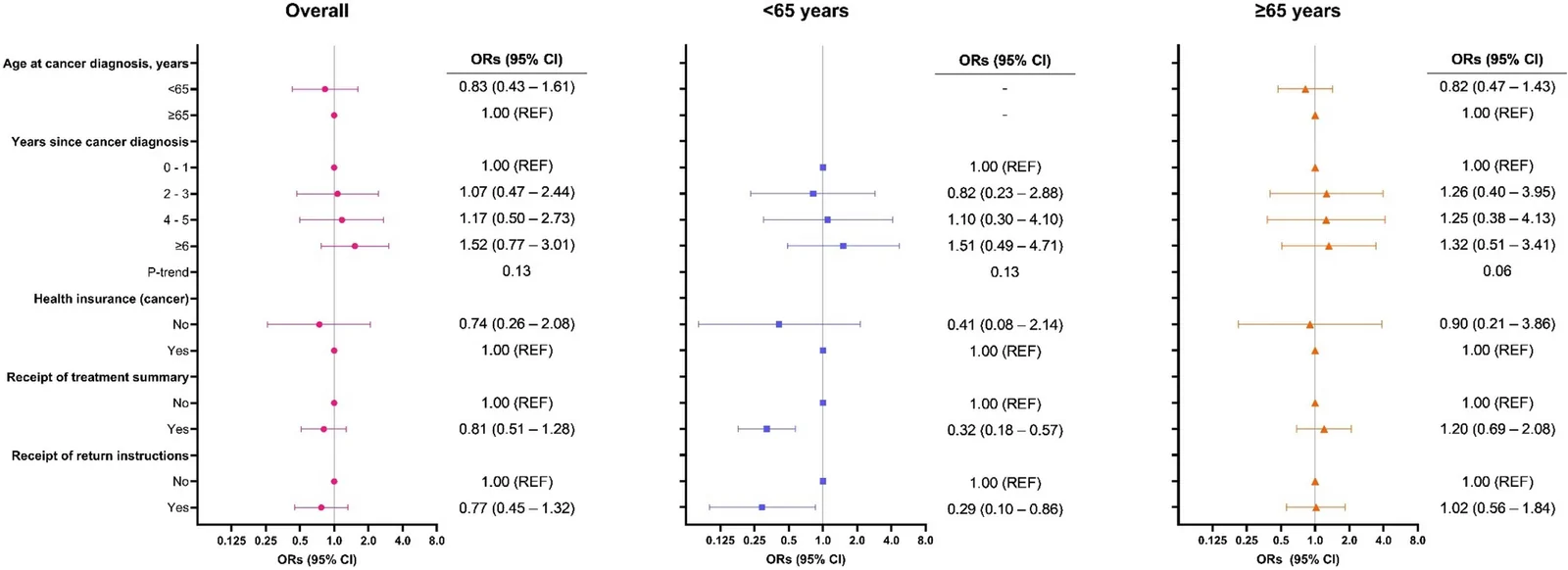
Associations between cancer survivorship factors and PCP-led survivorship care among breast cancer survivors, overall and stratified by age (n = 1,582)^a,b^. ^a^Models adjust for age (< 50, 50– < 60, 60– < 70, 70– < 80, ≥ 80 years), race and ethnicity (non-Hispanic White, non-Hispanic Black, non-Hispanic Asian, Hispanic, and non-Hispanic other race), educational attainment (high school or less, some college or technical school, college or technical school graduate), and annual income (< $25,000, $25,000–< $50,000, ≥ $50,000). ^b^Health insurance (cancer) was evaluated by asking participants whether they had health insurance that paid for all or part of their cancer treatment

Overall, associations between survivorship care factors and PCP-led care were not statistically significant (Fig. 3, Supplementary Table 7). However, among younger survivors, those who received a summary of their cancer treatments (OR: 0.32, 95% CI 0.18–0.57) and instructions regarding where to return for checkups after cancer treatment (OR: 0.29, 95% CI 0.10–0.86) had lower odds of PCP-led care compared with survivors who did not receive this information (Fig. 3). Although the odds of having PCP-led care increased with time since diagnosis, results were not statistically significant (2–3 years: OR: 1.07, 95% CI 0.47–2.44; 4–5 years: OR: 1.17, 95% CI 0.50–2.73; ≥ 6 years: OR: 1.52, 95% CI 0.77–3.01; P-trend: 0.13) (Fig. 3, Supplementary Table 7). Patterns of association were generally similar with restriction to participants with current health insurance coverage and when stratifying by time since diagnosis (Supplementary Table 8).

## Discussion

Our study comprehensively examined sociodemographic, lifestyle, clinical, and survivorship-related factors associated with PCP-led care among breast cancer survivors in the U.S. Overall, we found that breast cancer survivors who were NH-Black and NH-Asian, aged < 65 years, living in the South and West regions of the U.S., or had a history of myocardial infarction were less likely to engage with PCP-led care. Given the rising number of breast cancer survivors in the U.S., results from our study should heighten awareness to identify strategies to improve PCP-led care in breast cancer survivors, particularly among groups that may be less likely to engage in PCP-led care after cancer treatment.

### Age and PCP-led care

Consistent with prior literature, our study reported that older breast cancer survivors (age ≥ 65 years) were more likely to engage with primary care than younger women (aged < 65) [12, 35]. This finding may be due to a higher burden of comorbidities associated with increasing age as well as more common late and long-term effects of cancer treatment among older survivors [36, 37]. Differences in healthcare access due to Medicare eligibility at age 65 may also drive these findings. It is also plausible that younger women, particularly those aged < 50 years, may be receiving most of their care from other types of providers (e.g., obstetrician-gynecologists) or may be more likely to receive oncology-led care than PCP-led care due to a higher likelihood of being diagnosed with later stage disease and more aggressive subtypes. For example, compared to women aged ≥ 50 years, those aged < 50 were more likely to report oncology-led care (39.06% vs. 15.47%) and less likely to report PCP-led care (48.44% vs. 72.84%) in our study. Future research on PCP-led care among younger breast cancer survivors is warranted, particularly given differences in survivorship care for pre- vs. postmenopausal women as well as the rise of early-onset breast cancer over the past several decades [38].

### Race & ethnicity and PCP-led care

Adding to prior studies, our current study extends findings by reporting racial and ethnic differences in PCP-led care after breast cancer treatment. In our study, NH-Black and NH-Asian survivors were less likely to have PCP-led care compared to NH-White survivors. In contrast to our study, a prior study among breast cancer survivors found that NH-Black women were more likely to see a PCP than a medical oncologist when compared with NH-White women (OR: 2.62, 95% CI 1.47–4.65) [23]. Notably, this study examined follow-up care among recent survivors (at four years postdiagnosis), while our study primarily consisted of long-term survivors (≥ 6 years since diagnosis). Recommendations from ASCO suggest frequent follow-up visits with oncology providers within the first five years postdiagnosis [39]. These specific recommendations may explain differences between our results and the prior study. In addition, Black women are more likely to be diagnosed with triple-negative breast cancer, later-stage disease, and higher-grade tumors [1, 40], which may impact the need for oncology-led survivorship care. For example, Black women were more likely to report oncology-led care (33%) compared with White women (13%) in our study. This is consistent with a prior study that reported that minority women, including Black and Asian women, were more likely to prefer seeing an oncologist rather than PCP for their general preventive care [16]. In our study, Black women also had a slightly higher burden of comorbidities compared with White women (77% vs. 72% with one or more comorbid conditions); however, associations were unchanged when we adjusted for comorbidity burden in our multivariable models. The lower likelihood of PCP-led care among NH-Asian breast cancer survivors in our study may be driven by language barriers, cultural perceptions of cancer care, and a preference for oncology-led care in this group [41, 42]. Notably, we also reported that younger (< 65 years) Hispanic survivors were less likely to receive PCP-led care. It is also possible that language barriers may be a contributing factor to these findings, resulting in limited access to high-quality survivorship care [43]. Barriers related to these findings may also include transportation and financial concerns, lower health literacy, and limited social and emotional support [44]. Additional drivers for racial and ethnic differences in our study may include medical mistrust, systemic racism, structural barriers, and the multilevel interactions between race and other sociodemographic factors that influence healthcare access [45–47]. These findings highlight the need for future research on culturally tailored intervention strategies.

### Geographic region and PCP-led care

In our study, we found that breast cancer survivors living in the Southern and Western regions of the U.S. also had lower odds of receiving PCP-led care than those in the Midwest. It is plausible that migration may be driving these associations since the Southern and Western regions have significantly higher migration rates than those in the Midwest and the overall national rates [48]. Higher rates of migration may lead to fragmented care (e.g., frequent changes in healthcare providers) and may influence survivor disengagement with PCP-led care [49]. Differences in healthcare access among those living in rural and urban areas within these regions may also be driving these associations. Specifically, individuals living in rural communities experience greater barriers to healthcare access, including financial burden, geographic dispersion, and provider shortages, than those living in urban areas [50, 51]. Notably, our study population did not include more urban states such as California, Texas, or Florida since they did not opt in to include the BRFSS cancer survivorship module [27]. Their exclusion from our sample may have contributed to a higher prevalence of participants from more rural areas in the South and West. In rural areas, assistance with PCP-led care transitions may need to address transportation and geographic barriers, potentially incorporating more accessible methods such as telehealth [52].

### Heart disease and PCP-led care

In our study, we further found that breast cancer survivors with prior myocardial infarction were less likely to receive PCP-led care, particularly among younger and long-term survivors. Notably, breast cancer survivors have an increased risk of cardiovascular disease, which may be due to shared risk factors as well as cardiotoxic cancer treatment [5, 53, 54]. Breast cancer survivors with higher baseline cardiovascular risk and cardiovascular disease may also be more likely to receive close follow-up care from specialists (e.g., cardiologists) rather than primary care [55, 56]. In our study, although women who reported a history of coronary heart disease, angina, or stroke also had a lower likelihood of receiving PCP-led care, results were not statistically significant. Future larger studies are needed to confirm our findings and should further examine patterns of PCP-led vs. cardiology-led care and shared care models among breast cancer survivors with heart disease.

### Strengths and limitations

A strength of our study was the comprehensive approach for examining PCP-led care by including sociodemographic, lifestyle, clinical, and survivorship factors that leveraged the BRFSS data. Despite this strength, our study also has several limitations that should be considered. First, we were unable to include detailed cancer information, including breast cancer stage, subtype, and treatment, as well as information on subsequent cancers or treatment-related sequelae and other types of provider-led care (e.g., cardiology, obstetrics and gynecology), which may impact survivorship care and engagement with primary care. Second, results were self-reported in the BRFSS survey, including cancer history, and therefore may be susceptible to recall bias and misclassification. Third, we were unable to differentiate between types of health insurance, which may influence care practices, quality, and access. Lastly, although the BRFSS is a nationally representative survey, our findings might not be generalizable to states that did not include the survivorship module in their survey administration as well as male breast cancer survivors, and additional survivors that are underrepresented in the BRFSS data such as non-English speaking, immigrant, or undocumented survivors [57].

## Conclusion

With a growing number of breast cancer survivors in the U.S., survivorship care represents an increasing responsibility for healthcare systems across the nation. This growing burden is accompanied by a projected shortage in medical oncologists and oncology-specific health care in the upcoming decades, which raises concern over the ability for oncology teams to manage long-term survivorship care needs [58]. These trends highlight the importance to transition breast cancer survivors from oncology-led survivorship care to PCP-led or shared care models (e.g., onco-primary care) after the completion of cancer treatment. PCP-led care, as well as shared care models, are particularly important for the prevention and management of the late effects of cancer treatment, including controlling the burden of comorbidities. Our findings suggest that efforts to improve PCP-led care may need to be targeted toward specific groups that are less likely to report PCP-led care, including younger survivors, racial and ethnic minority survivors, and those with heart disease. Additional data regarding the perspectives of survivors from vulnerable groups and rural communities on their survivorship care would provide much needed guidance for developing effective interventions aimed at improving follow-up care for breast cancer survivors. Further studies are warranted to examine potential structural, geographic, and individual-level barriers to PCP-led care, particularly among younger survivors, racial and ethnic minorities, and those with heart disease, and to characterize region-specific differences with a focus on rural communities across the U.S.

## Supplementary Information

Below is the link to the electronic supplementary material.

Supplementary file1 (DOCX 123 KB)

## Data Availability

The data utilized in this study are publicly available from the United States Centers for Disease Control and Prevention at https://www.cdc.gov/brfss/annual_data/annual_data.htm.
