# Supplementary material for: Characterizing primary care in breast cancer survivors after completion of cancer therapy
Source: Cancer Causes Control. 2026 Aug 19;37(9):146. doi: 10.1007/s10552-026-02230-4 (PMC13489999; doi:10.1007/s10552-026-02230-4)
Supplement: Supplementary file 1 [file 10552_2026_2230_MOESM1_ESM.docx]

**SUPPLEMENTARY MATERIAL**

**Title:** Characterizing primary care in breast cancer survivors after completion of cancer therapy

**Journal Name:** *Cancer Causes and Control*

**Authors:** Kendall Kay, Yancen Pan, Jessica Li, Lan Zhang, Vanessa Torres, Yelba Castellon-Lopez, Meng-Han Tsai, Geetanjali Datta, Jennifer Henry, Cody Ramin

**Corresponding Author:**
Cody Ramin, PhD, SM
Cancer Research Center for Health Equity, Cedars-Sinai Medical Center, Los Angeles, CA, USA.
Cedars-Sinai Cancer, Cedars-Sinai Medical Center, Los Angeles, CA, USA.
[Cody.Ramin@csmc.edu](mailto:Cody.Ramin@cshs.org)

***Supplementary Figure 1. Identification of breast cancer survivors who completed active treatment among 108,639 respondents to the 2012, 2017, 2019, and 2021 BRFSS surveys.***

n = 2,285

Females with a breast cancer diagnosis

N = 108,639

Respondents to the BRFSS from states that administered the cancer survivorship module in 2012, 2017, 2019, or 2021

n = 1,613

Breast cancer survivors who completed cancer treatment

n = 31

Excluded those missing information on main survivorship care provider

n = 672

Excluded patients receiving active cancer treatment

n = 106,354

Excluded those without a breast cancer diagnosis (n=106,332) and males with a breast cancer diagnosis (n=22)

n = 1,582

Final analytic cohort of breast cancer survivors

***Supplementary Table 1. Overall characteristics of breast cancer survivors in the Behavioral Risk Factor Surveillance System by PCP-led survivorship care (n=1,582)***

| **Respondent characteristic** | **Non-PCP Led Care (n = 446)^a^** | | | | |  | **PCP Led Care (n = 1,136)** | | | | |
| --- | --- | --- | --- | --- | --- | --- | --- | --- | --- | --- | --- |
|  | **Unweighted** | |  | **Weighted** | |  | **Unweighted** | |  | **Weighted** | |
|  | **N** | **%** |  | **N** | **%** |  | **N** | **%** |  | **N** | **%** |
| Age, years |  |  |  |  |  |  |  |  |  |  |  |
| <50 | 33 | 7.40 |  | 4853 | 9.85 |  | 31 | 2.73 |  | 4459 | 4.06 |
| 50-<60 | 72 | 16.14 |  | 6859 | 13.92 |  | 117 | 10.30 |  | 14645 | 13.32 |
| 60-<70 | 128 | 28.70 |  | 15652 | 31.77 |  | 347 | 30.55 |  | 35147 | 31.97 |
| 70-<80 | 122 | 27.35 |  | 12933 | 26.25 |  | 398 | 35.04 |  | 36841 | 33.51 |
| ≥80 | 87 | 19.51 |  | 7966 | 16.17 |  | 235 | 20.69 |  | 17894 | 16.28 |
| No response | 4 | 0.90 |  | 998.49 | 2.03 |  | 8 | 0.70 |  | 958.13 | 0.87 |
| Age (binary), years |  |  |  |  |  |  |  |  |  |  |  |
| <65 | 156 | 34.98 |  | 20021 | 40.64 |  | 280 | 24.65 |  | 33795 | 30.74 |
| ≥65 | 286 | 64.13 |  | 28242 | 57.33 |  | 848 | 74.65 |  | 75191 | 68.39 |
| No response | 4 | 0.90 |  | 998.49 | 2.03 |  | 8 | 0.70 |  | 958.13 | 0.87 |
| Race & ethnicity |  |  |  |  |  |  |  |  |  |  |  |
| Non-Hispanic White | 325 | 72.87 |  | 32973 | 66.93 |  | 980 | 86.27 |  | 89507 | 81.41 |
| Non-Hispanic Black | 33 | 7.40 |  | 7022 | 14.25 |  | 44 | 3.87 |  | 7611 | 6.92 |
| Non-Hispanic Asian | 24 | 5.38 |  | 2277 | 4.62 |  | 32 | 2.82 |  | 2485 | 2.26 |
| Hispanic | 42 | 9.42 |  | 6148 | 12.48 |  | 36 | 3.17 |  | 8147 | 7.41 |
| Non-Hispanic other race | 22 | 4.93 |  | 841.36 | 1.71 |  | 44 | 3.87 |  | 2194 | 2.00 |
| Education level |  |  |  |  |  |  |  |  |  |  |  |
| High school or less | 139 | 31.17 |  | 19539 | 39.66 |  | 366 | 32.22 |  | 40041 | 36.42 |
| Some college/technical school | 152 | 34.08 |  | 18882 | 38.33 |  | 363 | 31.95 |  | 40462 | 36.80 |
| Graduated college/technical school | 155 | 34.75 |  | 10840 | 22.01 |  | 407 | 35.83 |  | 29440 | 26.78 |
| Annual income |  |  |  |  |  |  |  |  |  |  |  |
| <$25,000 | 116 | 26.01 |  | 14966 | 30.38 |  | 246 | 21.65 |  | 22407 | 20.38 |
| $25,000 - <$50,000 | 91 | 20.40 |  | 8717 | 17.70 |  | 303 | 26.67 |  | 25786 | 23.45 |
| ≥$50,000 | 155 | 34.75 |  | 16980 | 34.47 |  | 390 | 34.33 |  | 41071 | 37.36 |
| No response | 84 | 18.83 |  | 8598 | 17.45 |  | 197 | 17.34 |  | 20680 | 18.81 |
| Region |  |  |  |  |  |  |  |  |  |  |  |
| Midwest | 166 | 37.22 |  | 21848 | 44.35 |  | 704 | 61.97 |  | 70941 | 64.52 |
| South | 117 | 26.23 |  | 20389 | 41.39 |  | 139 | 12.24 |  | 25846 | 23.51 |
| West | 163 | 36.55 |  | 7024 | 14.26 |  | 293 | 25.79 |  | 13156 | 11.97 |
| Health insurance (current)**^b^** |  |  |  |  |  |  |  |  |  |  |  |
| No | 10 | 2.24 |  | 2271 | 4.61 |  | 19 | 1.67 |  | 3431 | 3.12 |
| Yes | 433 | 97.09 |  | 46832 | 95.07 |  | 1112 | 97.89 |  | 105811 | 96.24 |
| No response | 3 | 0.67 |  | 158.46 | 0.32 |  | 5 | 0.44 |  | 701.39 | 0.64 |
| BMI, kg/m^2^ |  |  |  |  |  |  |  |  |  |  |  |
| <25 | 158 | 35.43 |  | 15247 | 30.95 |  | 355 | 31.25 |  | 34265 | 31.17 |
| 25 - <30 | 146 | 32.74 |  | 21259 | 43.16 |  | 379 | 33.36 |  | 38908 | 35.39 |
| ≥30 | 111 | 24.89 |  | 10319 | 20.95 |  | 345 | 30.37 |  | 30381 | 27.63 |
| No response | 31 | 6.95 |  | 2437 | 4.95 |  | 57 | 5.02 |  | 6390 | 5.81 |
| Smoking status |  |  |  |  |  |  |  |  |  |  |  |
| Never smoked | 265 | 59.42 |  | 30625 | 62.17 |  | 693 | 61.00 |  | 67149 | 61.08 |
| Current/former smoker | 177 | 39.69 |  | 18477 | 37.51 |  | 438 | 38.56 |  | 42202 | 38.38 |
| No response | 4 | 0.90 |  | 159.76 | 0.32 |  | 5 | 0.44 |  | 593.33 | 0.54 |
| Alcohol consumption**^c^** |  |  |  |  |  |  |  |  |  |  |  |
| Non-excessive | 417 | 93.50 |  | 44323 | 89.98 |  | 1051 | 92.52 |  | 100250 | 91.18 |
| Excessive | 26 | 5.83 |  | 4777 | 9.70 |  | 79 | 6.95 |  | 8050 | 7.32 |
| No response | 3 | 0.67 |  | 161.65 | 0.33 |  | 6 | 0.53 |  | 1644 | 1.50 |
| Fruit & vegetable consumption |  |  |  |  |  |  |  |  |  |  |  |
| <5 times/day | 344 | 77.13 |  | 36904 | 74.92 |  | 862 | 75.88 |  | 83901 | 76.31 |
| ≥5 times/day | 75 | 16.82 |  | 9392 | 19.06 |  | 227 | 19.98 |  | 22729 | 20.67 |
| No response | 27 | 6.05 |  | 2966 | 6.02 |  | 47 | 4.14 |  | 3313 | 3.01 |
| Exercised in the past month**^d^** |  |  |  |  |  |  |  |  |  |  |  |
| No | 149 | 33.41 |  | 18196 | 36.94 |  | 335 | 29.49 |  | 32532 | 29.59 |
| Yes | 296 | 66.37 |  | 31017 | 62.96 |  | 800 | 70.42 |  | 77363 | 70.37 |
| No response | 1 | 0.22 |  | 48.08 | 0.10 |  | 1 | 0.09 |  | 48.01 | 0.04 |
| History of myocardial infarction |  |  |  |  |  |  |  |  |  |  |  |
| No | 411 | 92.15 |  | 44977 | 91.30 |  | 1075 | 94.63 |  | 105040 | 95.54 |
| Yes | 34 | 7.62 |  | 4122 | 8.37 |  | 59 | 5.19 |  | 4575 | 4.16 |
| No response | 1 | 0.22 |  | 162.65 | 0.33 |  | 2 | 0.18 |  | 328.17 | 0.30 |
| History of angina or CHD |  |  |  |  |  |  |  |  |  |  |  |
| No | 398 | 89.24 |  | 43177 | 87.65 |  | 1063 | 93.57 |  | 103115 | 93.79 |
| Yes | 40 | 8.97 |  | 4569 | 9.28 |  | 67 | 5.90 |  | 6598 | 6.00 |
| No response | 8 | 1.79 |  | 1516 | 3.08 |  | 6 | 0.53 |  | 230.49 | 0.21 |
| History of stroke |  |  |  |  |  |  |  |  |  |  |  |
| No | 423 | 94.84 |  | 46967 | 95.34 |  | 1058 | 93.13 |  | 102312 | 93.06 |
| Yes | 22 | 4.93 |  | 1965 | 3.99 |  | 77 | 6.78 |  | 7583 | 6.90 |
| No response | 1 | 0.22 |  | 329.51 | 0.67 |  | 1 | 0.09 |  | 48.04 | 0.04 |
| History of diabetes |  |  |  |  |  |  |  |  |  |  |  |
| No | 363 | 81.39 |  | 41322 | 83.88 |  | 929 | 81.78 |  | 91002 | 82.77 |
| Yes | 82 | 18.39 |  | 7756 | 15.74 |  | 207 | 18.22 |  | 18941 | 17.23 |
| No response | 1 | 0.22 |  | 184.18 | 0.37 |  | 0 | 0 |  | 0 | 0 |
| History of asthma |  |  |  |  |  |  |  |  |  |  |  |
| No | 377 | 84.53 |  | 40922 | 83.07 |  | 966 | 85.04 |  | 93094 | 84.67 |
| Yes | 68 | 15.25 |  | 8099 | 16.44 |  | 167 | 14.70 |  | 16156 | 14.70 |
| No response | 1 | 0.22 |  | 240.21 | 0.49 |  | 3 | 0.26 |  | 693.14 | 0.63 |
| History of COPD, emphysema, or chronic bronchitis |  |  |  |  |  |  |  |  |  |  |  |
| No | 397 | 89.01 |  | 43308 | 87.91 |  | 1012 | 89.08 |  | 99653 | 90.64 |
| Yes | 48 | 10.76 |  | 5762 | 11.70 |  | 119 | 10.48 |  | 10115 | 9.20 |
| No response | 1 | 0.22 |  | 191.63 | 0.39 |  | 5 | 0.44 |  | 175.52 | 0.16 |
| History of depressive disorder |  |  |  |  |  |  |  |  |  |  |  |
| No | 337 | 75.56 |  | 37274 | 75.67 |  | 867 | 76.32 |  | 78607 | 71.50 |
| Yes | 105 | 23.54 |  | 11758 | 23.87 |  | 266 | 23.42 |  | 31310 | 28.48 |
| No response | 4 | 0.90 |  | 188.78 | 0.47 |  | 3 | 0.26 |  | 26.86 | 0.02 |
| History of kidney disease |  |  |  |  |  |  |  |  |  |  |  |
| No | 406 | 91.03 |  | 45368 | 92.10 |  | 1057 | 93.05 |  | 103669 | 94.29 |
| Yes | 35 | 7.85 |  | 3442 | 6.99 |  | 76 | 6.69 |  | 6008 | 5.46 |
| No response | 5 | 1.12 |  | 452.37 | 0.92 |  | 3 | 0.26 |  | 266.76 | 0.24 |
| History of arthritis**^e^** |  |  |  |  |  |  |  |  |  |  |  |
| No | 248 | 55.61 |  | 22742 | 46.17 |  | 530 | 46.65 |  | 47229 | 42.96 |
| Yes | 196 | 43.95 |  | 26160 | 53.10 |  | 599 | 52.73 |  | 62240 | 56.61 |
| No response | 2 | 0.45 |  | 359.05 | 0.73 |  | 7 | 0.62 |  | 473.53 | 0.43 |
| History of one or more comorbid conditions**^f^** |  |  |  |  |  |  |  |  |  |  |  |
| No | 152 | 34.08 |  | 15018 | 30.49 |  | 285 | 25.09 |  | 25132 | 22.86 |
| Yes | 292 | 65.47 |  | 34208 | 69.44 |  | 842 | 74.12 |  | 84373 | 76.74 |
| No response | 2 | 0.45 |  | 35.98 | 0.07 |  | 9 | 0.79 |  | 438.26 | 0.40 |
| Age at cancer diagnosis (binary), years |  |  |  |  |  |  |  |  |  |  |  |
| <65 | 328 | 73.54 |  | 37053 | 75.22 |  | 823 | 72.45 |  | 80110 | 72.86 |
| ≥65 | 97 | 21.75 |  | 8836 | 17.94 |  | 288 | 25.35 |  | 26995 | 24.55 |
| No response | 21 | 4.71 |  | 3373 | 6.85 |  | 25 | 2.20 |  | 2839 | 2.58 |
| Years since cancer diagnosis |  |  |  |  |  |  |  |  |  |  |  |
| 0 – 1 | 38 | 8.52 |  | 3157 | 6.41 |  | 61 | 5.37 |  | 5366 | 4.88 |
| 2 – 3 | 53 | 11.88 |  | 4707 | 9.56 |  | 85 | 7.48 |  | 7841 | 7.13 |
| 4 – 5 | 38 | 8.52 |  | 4290 | 8.71 |  | 98 | 8.63 |  | 8682 | 7.90 |
| ≥6 | 290 | 65.02 |  | 32170 | 65.30 |  | 855 | 75.26 |  | 83947 | 76.35 |
| No response | 27 | 6.05 |  | 1404 | 10.02 |  | 37 | 3.26 |  | 4108 | 3.74 |
| Number of cancer diagnoses |  |  |  |  |  |  |  |  |  |  |  |
| One | 360 | 80.72 |  | 40414 | 82.04 |  | 948 | 83.45 |  | 94642 | 86.08 |
| Two | 71 | 15.92 |  | 7320 | 14.86 |  | 169 | 14.88 |  | 13983 | 12.72 |
| Three or more | 15 | 3.36 |  | 1527 | 3.10 |  | 19 | 1.67 |  | 1319 | 1.20 |
| Health insurance (cancer)**^g^** |  |  |  |  |  |  |  |  |  |  |  |
| No | 23 | 5.16 |  | 1980 | 4.02 |  | 42 | 3.70 |  | 3387 | 3.08 |
| Yes | 416 | 93.27 |  | 45144 | 91.64 |  | 1087 | 95.69 |  | 105772 | 96.21 |
| No response | 7 | 1.57 |  | 2139 | 4.34 |  | 7 | 0.62 |  | 784.55 | 0.71 |
| Receipt of treatment summary |  |  |  |  |  |  |  |  |  |  |  |
| No | 178 | 39.91 |  | 16954 | 34.42 |  | 516 | 45.42 |  | 45911 | 41.76 |
| Yes | 245 | 54.93 |  | 29627 | 60.14 |  | 554 | 48.77 |  | 60174 | 54.73 |
| No response | 23 | 5.16 |  | 2681 | 5.44 |  | 66 | 5.81 |  | 3858 | 3.51 |
| Receipt of return instructions |  |  |  |  |  |  |  |  |  |  |  |
| No | 77 | 17.26 |  | 8447 | 17.15 |  | 241 | 21.21 |  | 24901 | 22.65 |
| Yes | 360 | 80.72 |  | 39851 | 80.90 |  | 867 | 76.32 |  | 81678 | 74.29 |
| No response | 9 | 2.02 |  | 963.77 | 1.96 |  | 28 | 2.46 |  | 3364 | 3.06 |
| Abbreviations: BMI, Body Mass Index, CHD, Coronary Heart Disease | | | | | | | | | | | |
| **^a^**Non-PCP led care included oncology-led care defined as cancer surgeon, gynecologic oncologist, medical oncologist, radiation oncologist (58.5%), surgeon-led care defined as general surgeon, plastic or reconstructive surgeon (8.07%) and other-led care defined as urologist or other (33.4%). | | | | | | | | | | | |
| **^b^** Health insurance (current) was evaluated by asking participants if they had any form of healthcare coverage, prepaid plans such as HMOs, or government plans such as Medicare | | | | | | | | | | | |
| **^c^** Excessive alcohol consumption was defined as a daily average of more than one alcoholic drink or an episode of drinking four or more alcoholic drinks in the last month | | | | | | | | | | | |
| **^d^** Exercising in the past month includes 1 or more instance of doing any type of exercise in the past 30 days | | | | | | | | | | | |
| **^e^** History of arthritis is defined as arthritis, rheumatoid arthritis, gout, lupus, or fibromyalgia | | | | | | | | | | | |
| **^f^** History of one or more comorbid conditions is defined as having myocardial infarction, angina or coronary heart disease, stroke, asthma, chronic obstructive pulmonary disease, emphysema, chronic bronchitis, depressive disorder, kidney disease, diabetes, arthritis, rheumatoid arthritis, gout, lupus, or fibromyalgia | | | | | | | | | | | |
| **^g^** Health insurance (cancer) was evaluated by asking participants whether they had health insurance that paid for all or part of their cancer treatment | | | | | | | | | | | |

***Supplementary Table 2. Association between sociodemographic factors and PCP-led survivorship care among breast cancer survivors (n=1,582)***

| **Sociodemographic factors** | **Model 1^a^** | |  | **Model 2^b^** | |  | **Model 3^c^** | |
| --- | --- | --- | --- | --- | --- | --- | --- | --- |
|  | **OR** | **95% CI** |  | **OR** | **95% CI** |  | **OR** | **95% CI** |
| Age, years |  |  |  |  |  |  |  |  |
| <50 | 0.41 | 0.15 – 1.10 |  | 0.46 | 0.19 – 1.10 |  | 0.53 | 0.22 – 1.28 |
| 50-<60 | 0.95 | 0.48 – 1.87 |  | 0.96 | 0.49 – 1.88 |  | 0.99 | 0.50 – 1.93 |
| 60-<70 | 1.00 | 0.55 – 1.81 |  | 0.99 | 0.54 – 1.83 |  | 1.00 | 0.54 – 1.84 |
| 70-<80 | 1.27 | 0.69 – 2.34 |  | 1.41 | 0.74 – 2.67 |  | 1.44 | 0.76 – 2.73 |
| ≥80 | 1.00 | (Ref) |  | 1.00 | (Ref) |  | 1.00 | (Ref) |
| P-trend | 0.10 | |  | 0.09 | |  | 0.17 | |
| Age (binary), years |  |  |  |  |  |  |  |  |
| <65 | 0.63 | 0.42 – 0.97 |  | 0.63 | 0.41 – 0.98 |  | 0.65 | 0.42 – 1.01 |
| ≥65 | 1.00 | (Ref) |  | 1.00 | (Ref) |  | 1.00 | (Ref) |
| Race & ethnicity |  |  |  |  |  |  |  |  |
| Non-Hispanic White | 1.00 | (Ref) |  | 1.00 | (Ref) |  | 1.00 | (Ref) |
| Non-Hispanic Black | 0.44 | 0.21 – 0.90 |  | 0.49 | 0.24 – 0.97 |  | 0.50 | 0.25 – 1.01 |
| Non-Hispanic Asian | 0.38 | 0.14 – 1.07 |  | 0.33 | 0.11 – 0.97 |  | 0.33 | 0.11 – 0.97 |
| Hispanic | 0.48 | 0.21 – 1.12 |  | 0.57 | 0.23 – 1.37 |  | 0.59 | 0.25 – 1.42 |
| Non-Hispanic other race | 0.98 | 0.34 – 2.82 |  | 1.08 | 0.35 – 3.30 |  | 1.27 | 0.33 – 4.89 |
| Education level |  |  |  |  |  |  |  |  |
| High school or less | 0.60 | 0.38 – 0.96 |  | 0.69 | 0.42 – 1.12 |  | 0.71 | 0.44 – 1.14 |
| Some college/technical school | 0.69 | 0.44 – 1.10 |  | 0.67 | 0.42 – 1.08 |  | 0.69 | 0.43 – 1.11 |
| Graduated college/technical school | 1.00 | (Ref) |  | 1.00 | (Ref) |  | 1.00 | (Ref) |
| Annual income |  |  |  |  |  |  |  |  |
| <$25,000 | 0.58 | 0.33 – 1.03 |  | 0.73 | 0.40 – 1.33 |  | 0.75 | 0.41 – 1.37 |
| $25,000 - <$50,000 | 1.11 | 0.65 – 1.90 |  | 1.17 | 0.66 – 2.08 |  | 1.20 | 0.68 – 2.11 |
| ≥$50,000 | 1.00 | (Ref) |  | 1.00 | (Ref) |  | 1.00 | (Ref) |
| P-trend | 0.07 | |  | 0.36 | |  | 0.45 | |
| Region |  |  |  |  |  |  |  |  |
| Midwest | 1.00 | (Ref) |  | 1.00 | (Ref) |  | 1.00 | (Ref) |
| South & territories | 0.38 | 0.23 – 0.63 |  | 0.42 | 0.25 – 0.72 |  | 0.42 | 0.25 – 0.72 |
| West | 0.56 | 0.37 – 0.84 |  | 0.60 | 0.37 – 0.96 |  | 0.60 | 0.37 – 0.96 |
| **^a^** Model 1 adjusted for age (<50, 50-<60, 60-<70, 70-<80, ≥80 years) | | | | | | | | |
| **^b^** Model 2 adjusted for Model 1 factors, as well as race and ethnicity (non-Hispanic White, non-Hispanic Black, non-Hispanic Asian, Hispanic, and non-Hispanic other race), education level (did not graduate/graduate high school, attended college/technical school, graduated college/technical school), annual income (<$25,000, $25,000 - <$50,000, ≥$50,000) | | | | | | | | |
| **^c^** Model 3 adjusted for all Model 2 factors, as well as health insurance during cancer treatment (yes, no) | | | | | | | | |

***Supplementary Table 3. Association between sociodemographic factors and PCP-led survivorship care among breast cancer survivors 1) restricted to those with health insurance at time of survey (n=1,545) and 2) stratified by time since diagnosis*^a^**

| **Sociodemographic factors** | **Restricted to those with current health insurance *(n=1,545)*** | |  | **Stratified by time since diagnosis** | | | |
| --- | --- | --- | --- | --- | --- | --- | --- |
|  |  |  |  | **<5 years (n = 292)** | | **≥5 years (n = 1,226)** | |
|  | **OR** | **95% CI** |  | **OR** | **95% CI** | **OR** | **95% CI** |
| Race & ethnicity |  |  |  |  |  |  |  |
| Non-Hispanic White | 1.00 | (Ref) |  | 1.00 | (Ref) | 1.00 | (Ref) |
| Non-Hispanic Black | 0.47 | 0.24 – 0.90 |  | 0.32 | 0.12 – 0.85 | 0.66 | 0.29 – 1.49 |
| Non-Hispanic Asian | 0.35 | 0.12 – 1.04 |  | 0.04 | 0.01 – 0.16 | 0.63 | 0.27 – 1.46 |
| Hispanic | 0.61 | 0.25 – 1.50 |  | 0.19 | 0.05 – 0.71 | 0.62 | 0.20 – 1.91 |
| Non-Hispanic other race | 0.81 | 0.31 – 2.15 |  | 2.24 | 0.42 – 11.92 | 1.04 | 0.26 – 4.08 |
| Education level |  |  |  |  |  |  |  |
| High school or less | 0.66 | 0.41 – 1.06 |  | 0.97 | 0.40 – 2.35 | 0.72 | 0.40 – 1.28 |
| Some college/technical school | 0.72 | 0.45 – 1.15 |  | 1.19 | 0.55 – 2.55 | 0.56 | 0.33 – 0.96 |
| Graduated college/technical school | 1.00 | (Ref) |  | 1.00 | (Ref) | 1.00 | (Ref) |
| Annual income |  |  |  |  |  |  |  |
| <$25,000 | 0.67 | 0.37 – 1.23 |  | 0.76 | 0.31 – 1.86 | 0.86 | 0.42 – 1.73 |
| $25,000 - <$50,000 | 1.16 | 0.66 – 2.05 |  | 1.77 | 0.69 – 4.50 | 1.13 | 0.58 – 2.20 |
| ≥$50,000 | 1.00 | (Ref) |  | 1.00 | (Ref) | 1.00 | (Ref) |
| P-trend | 0.44 |  |  | 0.89 |  | 0.65 |  |
| Region |  |  |  |  |  |  |  |
| Midwest | 1.00 | (Ref) |  | 1.00 | (Ref) | 1.00 | (Ref) |
| South | 0.42 | 0.24 – 0.72 |  | 0.35 | 0.14 – 0.84 | 0.40 | 0.22 – 0.73 |
| West | 0.63 | 0.39 – 1.02 |  | 0.62 | 0.24 – 1.60 | 0.52 | 0.32 – 0.84 |
| **^a^** Model adjusted for age (<50, 50-<60, 60-<70, 70-<80, ≥80 years), race and ethnicity (non-Hispanic White, non-Hispanic Black, non-Hispanic Asian, Hispanic, and non-Hispanic other race), education level (did not graduate/graduate high school, attended college/technical school, graduated college/technical school), annual income (<$25,000, $25,000 - <$50,000, ≥$50,000) | | | | | | | |

***Supplementary Table 4. Association between lifestyle and clinical factors and PCP-led survivorship care among breast cancer survivors (n=1,582)***

| **Lifestyle and clinical factors** | **Model 1^a^** | |  | **Model 2^b^** | |  | **Model 3^c^** | |
| --- | --- | --- | --- | --- | --- | --- | --- | --- |
|  | **OR** | **95% CI** |  | **OR** | **95% CI** |  | **OR** | **95% CI** |
| BMI, kg/m^2^ |  |  |  |  |  |  |  |  |
| <25 | 1.00 | (Ref) |  | 1.00 | (Ref) |  | 1.00 | (Ref) |
| 25 - <30 | 0.78 | 0.47 – 1.30 |  | 0.83 | 0.49 – 1.41 |  | 0.85 | 0.50 – 1.44 |
| ≥30 | 1.32 | 0.78 – 2.22 |  | 1.27 | 0.73 – 2.20 |  | 1.28 | 0.74 – 2.22 |
| P-trend | 0.33 | |  | 0.42 | |  | 0.39 | |
| Smoking status |  |  |  |  |  |  |  |  |
| Never smoked | 1.00 | (Ref) |  | 1.00 | (Ref) |  | 1.00 | (Ref) |
| Current/former smoker | 1.00 | 0.66 – 1.51 |  | 0.99 | 0.64 – 1.54 |  | 1.01 | 0.65 – 1.56 |
| Alcohol consumption**^d^** |  |  |  |  |  |  |  |  |
| Non-excessive | 1.00 | (Ref) |  | 1.00 | (Ref) |  | 1.00 | (Ref) |
| Excessive | 0.70 | 0.28 – 1.77 |  | 0.63 | 0.26 – 1.56 |  | 0.63 | 0.26 – 1.55 |
| Fruit and vegetable consumption |  |  |  |  |  |  |  |  |
| <5 times/day | 0.91 | 0.51 – 1.60 |  | 1.06 | 0.61 – 1.86 |  | 1.06 | 0.60 – 1.86 |
| ≥5 times/day | 1.00 | (Ref) |  | 1.00 | (Ref) |  | 1.00 | (Ref) |
| Exercised in the past month**^e^** |  |  |  |  |  |  |  |  |
| No | 0.68 | 0.45 – 1.04 |  | 0.75 | 0.47 – 1.19 |  | 0.76 | 0.48 – 1.19 |
| Yes | 1.00 | (Ref) |  | 1.00 | (Ref) |  | 1.00 | (Ref) |
| History of myocardial infarction |  |  |  |  |  |  |  |  |
| No | 1.00 | (Ref) |  | 1.00 | (Ref) |  | 1.00 | (Ref) |
| Yes | 0.46 | 0.23 – 0.90 |  | 0.40 | 0.20 – 0.80 |  | 0.40 | 0.20 – 0.80 |
| History of diabetes |  |  |  |  |  |  |  |  |
| No | 1.00 | (Ref) |  | 1.00 | (Ref) |  | 1.00 | (Ref) |
| Yes | 1.03 | 0.64 – 1.65 |  | 1.14 | 0.67 – 1.93 |  | 1.15 | 0.68 – 1.94 |
| Abbreviations: BMI, Body Mass Index | | | | | | | | |
| **^a^** Model 1 adjusted for age (<50, 50-<60, 60-<70, 70-<80, ≥80 years) | | | | | | | | |
| **^b^** Model 2 adjusted for Model 1 factors, as well as race and ethnicity (non-Hispanic White, non-Hispanic Black, non-Hispanic Asian, Hispanic, and non-Hispanic other race), education level (did not graduate/graduate high school, attended college/technical school, graduated college/technical school), annual income (<$25,000, $25,000 - <$50,000, ≥$50,000) | | | | | | | | |
| **^c^** Model 3 adjusted for all Model 2 factors, as well as health insurance during cancer treatment (yes, no) | | | | | | | | |
| **^d^** Excessive alcohol consumption was defined as a daily average of more than one alcoholic drink or an episode of drinking four or more alcoholic drinks in the last month | | | | | | | | |
| **^e^** Exercising in the past month includes 1 or more instance of doing any type of exercise in the past 30 days | | | | | | | | |

***Supplementary Table 5. Association between additional comorbidities and PCP-led survivorship care among breast cancer survivors (n=1,582)*^a^**

| **Comorbid condition** | **OR** | **95% CI** |
| --- | --- | --- |
| History of one or more comorbid conditions**^c^** |  |  |
| Yes | 1.46 | 0.95 – 2.25 |
| No | 1.00 | (REF) |
| History of angina or CHD |  |  |
| Yes | 0.53 | 0.22 – 1.30 |
| No | 1.00 | (REF) |
| History of stroke |  |  |
| Yes | 1.69 | 0.77 – 3.70 |
| No | 1.00 | (REF) |
| History of asthma |  |  |
| Yes | 0.94 | 0.54 – 1.63 |
| No | 1.00 | (REF) |
| History of COPD, emphysema, or chronic bronchitis |  |  |
| Yes | 0.74 | 0.38 – 1.43 |
| No | 1.00 | (REF) |
| History of depressive disorder |  |  |
| Yes | 1.32 | 0.79 – 2.21 |
| No | 1.00 | (REF) |
| History of kidney disease |  |  |
| Yes | 0.64 | 0.34 – 1.22 |
| No | 1.00 | (REF) |
| History of arthritis**^b^** |  |  |
| Yes | 1.07 | 0.72 – 1.59 |
| No | 1.00 | (REF) |
| Abbreviations: CHD, coronary heart disease; COPD, chronic obstructive pulmonary disease | | |
| **^a^** Model adjusted for age (<50, 50-<60, 60-<70, 70-<80, ≥80 years), race and ethnicity (non-Hispanic White, non-Hispanic Black, non-Hispanic Asian, Hispanic, and non-Hispanic other race), education level (did not graduate/graduate high school, attended college/technical school, graduated college/technical school), annual income (<$25,000, $25,000 - <$50,000, ≥$50,000) | | |
| **^b^** History of arthritis is defined as having been told one had arthritis, rheumatoid arthritis, gout, lupus, or fibromyalgia | | |
| **^c^** History of one or more comorbid conditions is defined as myocardial infarction, angina or coronary heart disease, stroke, asthma, chronic obstructive pulmonary disease, emphysema, chronic bronchitis, depressive disorder, kidney disease, diabetes, arthritis, rheumatoid arthritis, gout, lupus, or fibromyalgia | | |

***Supplementary Table 6. Association between lifestyle, clinical factors, and PCP-led survivorship care among breast cancer survivors 1) restricted to those with health insurance at time of survey (n = 1,545) and 2) stratified by time since diagnosis*^a^**

| **Lifestyle and clinical factors** | **Restricted to those with current health insurance *(n=1,545)*** | |  | **Stratified by time since diagnosis** | | | |
| --- | --- | --- | --- | --- | --- | --- | --- |
|  |  |  |  | **<5 years (n = 292)** | | **≥5 years (n = 1,226)** | |
|  | **OR** | **95% CI** |  | **OR** | **95% CI** | **OR** | **95% CI** |
| BMI, kg/m^2^ |  |  |  |  |  |  |  |
| <25 | 1.00 | (Ref) |  | 1.00 | (Ref) | 1.00 | (Ref) |
| 25 - <30 | 0.90 | 0.53 – 1.53 |  | 1.67 | 0.55 – 5.06 | 0.75 | 0.42 – 1.33 |
| ≥30 | 1.33 | 0.77 – 2.27 |  | 2.03 | 0.84 – 4.89 | 1.15 | 0.60 – 2.19 |
| P-trend | 0.64 | |  | 0.11 | | 0.67 | |
| Smoking status |  |  |  |  |  |  |  |
| Never smoked | 1.00 | (Ref) |  | 1.00 | (Ref) | 1.00 | (Ref) |
| Current/former smoker | 0.89 | 0.58 – 1.37 |  | 0.49 | 0.24 – 1.01 | 1.07 | 0.64 – 1.76 |
| Alcohol consumption**^b^** |  |  |  |  |  |  |  |
| Non-excessive | 1.00 | (Ref) |  | 1.00 | (Ref) | 1.00 | (Ref) |
| Excessive | 0.62 | 0.25 – 1.54 |  | 7.86 | 1.79 – 34.46 | 0.50 | 0.17 – 1.42 |
| Fruit and vegetable consumption |  |  |  |  |  |  |  |
| <5 times/day | 1.14 | 0.65 – 2.00 |  | 0.22 | 0.10 – 0.50 | 1.32 | 0.69 – 2.51 |
| ≥5 times/day | 1.00 | (Ref) |  | 1.00 | (Ref) | 1.00 | (Ref) |
| Exercised in the past month**^c^** |  |  |  |  |  |  |  |
| No | 0.78 | 0.49 – 1.25 |  | 0.39 | 0.17 – 0.89 | 0.73 | 0.44 – 1.21 |
| Yes | 1.00 | (Ref) |  | 1.00 | (Ref) | 1.00 | (Ref) |
| History of myocardial infarction |  |  |  |  |  |  |  |
| No | 1.00 | (Ref) |  | 1.00 | (Ref) | 1.00 | (Ref) |
| Yes | 0.42 | 0.21 – 0.86 |  | 1.80 | 0.60 – 5.40 | 0.27 | 0.13 – 0.59 |
| History of diabetes |  |  |  |  |  |  |  |
| No | 1.00 | (Ref) |  | 1.00 | (Ref) | 1.00 | (Ref) |
| Yes | 1.23 | 0.72 – 2.11 |  | 1.91 | 0.89 – 4.11 | 0.93 | 0.52 – 1.66 |
| Abbreviations: BMI, Body Mass Index | | | | | | | |
| **^a^** Model adjusted for age (<50, 50-<60, 60-<70, 70-<80, ≥80 years), race and ethnicity (non-Hispanic White, non-Hispanic Black, non-Hispanic Asian, Hispanic, and non-Hispanic other race), education level (did not graduate/graduate high school, attended college/technical school, graduated college/technical school), annual income (<$25,000, $25,000 - <$50,000, ≥$50,000) | | | | | | | |
| **^b^** Excessive alcohol consumption was defined as a daily average of more than one alcoholic drink or an episode of drinking four or more alcoholic drinks in the last month | | | | | | | |
| **^c^** Exercising in the past month includes 1 or more instance of doing any type of exercise in the past 30 days | | | | | | | |

***Supplementary Table 7. Association between cancer survivorship factors and PCP-led survivorship care among breast cancer survivors (n=1,582)***

| **Cancer survivorship factors** | **Model 1^a^** | |  | **Model 2^b^** | |  | **Model 3^c^** | |
| --- | --- | --- | --- | --- | --- | --- | --- | --- |
|  | **OR** | **95% CI** |  | **OR** | **95% CI** |  | **OR** | **95% CI** |
| Age at cancer diagnosis, years |  |  |  |  |  |  |  |  |
| <65 | 0.85 | 0.47 – 1.55 |  | 0.83 | 0.43 – 1.61 |  | 0.82 | 0.43 – 1.58 |
| ≥65 | 1.00 | (Ref) |  | 1.00 | (Ref) |  | 1.00 | (Ref) |
| Years since cancer diagnosis |  |  |  |  |  |  |  |  |
| 0 – 1 | 1.00 | (Ref) |  | 1.00 | (Ref) |  | 1.00 | (Ref) |
| 2 – 3 | 0.97 | 0.42 – 2.26 |  | 1.07 | 0.47 – 2.44 |  | 1.06 | 0.47 – 2.38 |
| 4 – 5 | 1.13 | 0.45 – 2.81 |  | 1.17 | 0.50 – 2.73 |  | 1.15 | 0.50 – 2.67 |
| ≥6 | 1.44 | 0.74 – 2.82 |  | 1.52 | 0.77 – 3.01 |  | 1.53 | 0.78 – 2.98 |
| P-trend | 0.13 | |  | 0.13 | |  | 0.14 | |
| Health insurance (cancer)**^d^** |  |  |  |  |  |  |  |  |
| No | 0.69 | 0.27 – 1.78 |  | 0.74 | 0.26 – 2.08 |  | 0.74 | 0.26 – 2.08 |
| Yes | 1.00 | (Ref) |  | 1.00 | (Ref) |  | 1.00 | (Ref) |
| Receipt of treatment summary |  |  |  |  |  |  |  |  |
| No | 1.00 | (Ref) |  | 1.00 | (Ref) |  | 1.00 | (Ref) |
| Yes | 0.78 | 0.51 – 1.21 |  | 0.81 | 0.51 – 1.28 |  | 0.79 | 0.50 – 1.26 |
| Receipt of return instructions |  |  |  |  |  |  |  |  |
| No | 1.00 | (Ref) |  | 1.00 | (Ref) |  | 1.00 | (Ref) |
| Yes | 0.75 | 0.44 – 1.27 |  | 0.77 | 0.45 – 1.32 |  | 0.76 | 0.44 – 1.30 |
| **^a^** Model 1 adjusts for age (<50, 50-<60, 60-<70, 70-<80, ≥80 years) | | | | | | | | |
| **^b^** Model 2 adjusts for Model 1 factors, as well as race and ethnicity (non-Hispanic White, non-Hispanic Black, non-Hispanic Asian, Hispanic, and non-Hispanic other race), education level (did not graduate/graduate high school, attended college/technical school, graduated college/technical school), annual income (<$25,000, $25,000 - <$50,000, ≥$50,000) | | | | | | | | |
| **^c^** Model 3 adjusts for all Model 2 covariates, as well as health insurance (cancer) (yes, no) | | | | | | | | |
| **^d^** Health insurance (cancer) was evaluated by asking participants whether they had health insurance that paid for all or part of their cancer treatment | | | | | | | | |

***Supplementary Table 8. Association between cancer survivorship factors and PCP-led survivorship care among breast cancer survivors 1) restricted to those with health insurance at time of survey (n=1,545) and 2) stratified by time since diagnosis*^a^**

| **Cancer survivorship factors** | **Restricted to those with current health insurance *(n=1,545)*** | |  | **Stratified by time since diagnosis** | | | |
| --- | --- | --- | --- | --- | --- | --- | --- |
|  |  |  |  | **<5 years (n = 292)** | | **≥5 years (n = 1,226)** | |
|  | **OR** | **95% CI** |  | **OR** | **95% CI** | **OR** | **95% CI** |
| Age at cancer diagnosis, years |  |  |  |  |  |  |  |
| <65 | 0.83 | 0.43 – 1.61 |  | 1.04 | 0.32 – 3.39 | 0.56 | 0.24 – 1.28 |
| ≥65 | 1.00 | (Ref) |  | 1.00 | (Ref) | 1.00 | (Ref) |
| Years since cancer diagnosis |  |  |  |  |  |  |  |
| 0 – 1 | 1.00 | (Ref) |  | 1.00 | (Ref) | 1.00 | (Ref) |
| 2 – 3 | 1.09 | 0.48 – 2.45 |  | - | - | - | - |
| 4 – 5 | 1.23 | 0.53 – 2.86 |  | - | - | - | - |
| ≥6 | 1.53 | 0.77 – 3.01 |  | - | - | - | - |
| P-trend | 0.34 | |  | - | - | - | - |
| Health insurance (cancer)**^b^** |  |  |  |  |  |  |  |
| No | 0.95 | 0.29 – 3.15 |  | 0.13 | 0.01 – 1.39 | 1.06 | 0.43 – 2.65 |
| Yes | 1.00 | (Ref) |  | 1.00 | (Ref) | 1.00 | (Ref) |
| Receipt of treatment summary |  |  |  |  |  |  |  |
| No | 1.00 | (Ref) |  | 1.00 | (Ref) | 1.00 | (Ref) |
| Yes | 0.83 | 0.52 – 1.32 |  | 1.52 | 0.67 – 3.45 | 0.76 | 0.46 – 1.27 |
| Receipt of return instructions |  |  |  |  |  |  |  |
| No | 1.00 | (Ref) |  | 1.00 | (Ref) | 1.00 | (Ref) |
| Yes | 0.76 | 0.45 – 1.31 |  | 0.83 | 0.35 – 1.95 | 0.68 | 0.38 – 1.22 |
| **^a^** Model adjusted for age (<50, 50-<60, 60-<70, 70-<80, ≥80 years), race and ethnicity (non-Hispanic White, non-Hispanic Black, non-Hispanic Asian, Hispanic, and non-Hispanic other race), education level (did not graduate/graduate high school, attended college/technical school, graduated college/technical school), annual income (<$25,000, $25,000 - <$50,000, ≥$50,000)  **^b^** Health insurance (cancer) was evaluated by asking participants whether they had health insurance that paid for all or part of their cancer treatment | | | | | | | |
